# Airborne imagery does not preclude detectability issues in estimating bird colony size

**DOI:** 10.1038/s41598-024-53961-w

**Published:** 2024-02-14

**Authors:** Thibaut Couturier, Laurie Gaillard, Almodis Vadier, Emilien Dautrey, Jérôme Mathey, Aurélien Besnard

**Affiliations:** 1 CEFE, IRD, CNRS, University of Montpellier, EPHE-PSL University, Montpellier, France; 2 GEPOMAY, Groupe d’Études et de Protection des Oiseaux de Mayotte, 4 Impasse Tropina, Miréréni, Tsingoni, Mayotte France; 3 DroneGo, Quartier Hadoume, Bp33 Poste de Combani, Tsingoni, Mayotte France

**Keywords:** Biodiversity, Ecological modelling, Population dynamics

## Abstract

Aerial images obtained by drones are increasingly used for ecological research such as wildlife monitoring. Yet detectability issues resulting from animal activity or visibility are rarely considered, although these may lead to biased population size and trend estimates. In this study, we investigated detectability in a census of Malagasy pond heron *Ardeola idae* colonies on the island of Mayotte. We conducted repeated drone flights over breeding colonies in mangrove habitats during two breeding seasons. We then identified individuals and nests in the images and fitted closed capture-recapture models on nest-detection histories. We observed seasonal variation in the relative abundance of individuals, and intra-daily variation in the relative abundance of individuals—especially immature birds—affecting the availability of nests for detection. The detection probability of nests estimated by capture–recapture varied between 0.58 and 0.74 depending on flyover days and decreased 25% from early to late morning. A simulation showed that three flyovers are necessary to detect a 5–6% decline in colonies of 50 to 200 nests. These results indicate that the detectability of nests of forest-canopy breeding species from airborne imagery can vary over space and time; we recommend the use of capture-recapture methods to control for this bias.

## Introduction

Aerial images obtained by unmanned aerial vehicles (UAVs, or drones) are revolutionizing data collection in ecology, providing information on the presence, abundance and behaviour of animals at high spatial resolution^1,2^. This survey method is particularly useful for monitoring gregarious species, as it reportedly causes less disturbance, increases count accuracy and provides a better assessment of the age and sex composition of groups than other conventional approaches such as ground or sea surveys^3,4^. Drones are thus being increasingly used for the population monitoring of many vertebrate species, such as crocodiles^5^, chimpanzees^6^, marine mammals^7^, and birds^8^, notably for bird species breeding in colonies, either on the ground^9,10^ or in the canopy^11,12^. Aerial images of birds can be used to provide estimates of breeding success^11–13^ and abundance of nesting individuals at a higher accuracy than ground observations^14^.

Yet as in a conventional census, counts of individual birds or nests obtained from airborne imagery may be subject to detectability issues^15^. Overall detection probability from an aerial census is the product of three components of the detection process: (1) probability that an individual bird associated with the sampling area is present during the count (i.e. presence/absence of the individual in the colony when the flyover is performed), (2) probability that an individual is available for detection (i.e. individual visible or hidden at the time of image capture) given it is present in the sampling area, and (3) probability that an individual is detected by the image interpreter given it is present and available for detection^16,17^.

In colonial breeding birds, not all breeding individuals are simultaneously present in a colony: for example, they may need to forage outside the colony. This would lead to the detection probability of the first component of the detection process to be below one. The proportion of individuals present may also vary over time: for example, due to circadian rhythm^18^ or species phenology^19^, leading to heterogeneous detection probability. For example, high variation in nest attendance rates between the incubation and chick-rearing stages have been observed in colonial birds^18,20,21^.

In addition, some individuals or nests may be completely hidden by vegetation cover or terrain. This may be the case when located under trees for canopy-nesting species^15^, or under rock ledges for cliff-nesting species^22^. This would result in the bird or nest being unavailable for detection in aerial images even when present, impacting the second component of the detection process.

Lastly, even when individuals are present and available for detection, various phenomena can affect the third component of the detection process when using aerial images: detection by the image interpreter^15,21,23^. Many of these detection issues are identical to those encountered in visual aerial surveys^24,25^. For example, a lack of contrast between birds and the substrate or vegetation or weather and lighting conditions may affect the detectability of individuals and/or nests by image interpreters^15,21^. The visibility of individuals or nests can also depend on the species’ body size, traits and behaviour^10,15,26,27^. Species at high risk of being misidentified as another species may be prone to detection issues^23,26^. Image resolution can influence the detectability of individuals^21^ or objects like nests^6^. And just as with direct observation in the field, the image interpreter’s level of experience, visual acuity, weariness etc. can also strongly affect detection ability^25,28,29^.

If not taken into account, these detectability issues may lead to biased abundance estimates and their variation in time and space^25,30^. This has been identified as a problem in population monitoring literature over the last five decades, resulting in the development of several methods (such as capture-recapture^31^ and distance sampling^32^) to explicitly model detection probability in order to provide unbiased estimates of the true abundance of individuals. Traditional capture-recapture methods require animals to be individually identified. While individual identification from aerial images may sometimes be possible for mammals^33^, this is rarely possible for bird species, which typically need to be captured and individually marked. This can be a very complex and time-consuming task for colonial bird species, and it can also result in high disturbance of breeders^34,35^. Moreover, mark resights are usually not possible from aerial images.

An alternative to marking individuals is ‘double counting’, or the use of the Lincoln-Petersen index^36^. This technique, based on similar reasoning to capture-recapture, has been used for decades to estimate bird abundance from aerial surveys^24,37^, and more recently from airborne imagery^15^. It consists of counting the individuals independently detected by a primary observer, a secondary observer and both observers (or on the same image)^38^. Yet such an approach only takes into account the third component of the detection process (detection probability by an image interpreter when an individual is present and available for detection), assuming the other two components are equal to one. This assumption is problematic if the goal is to estimate absolute abundance, or to compare relative abundance between sites^17^, or over time.

Ideally, a method should take into account all three components of the detection process, as in traditional capture-recapture; yet such a method has never been used for estimating bird abundance from aerial surveys. In theory, an aerial census should be relevant for estimating the abundance of fixed objects such as nests, by geolocating them on sequential aerial images. However, repeated drone flights over a colony may increase disturbance risks^35,39,40^. It is thus crucial to determine the optimal sampling effort to get an accurate estimate of abundance with a minimal number of flyovers. This study aimed to assess this and to test the reliability of the capture-recapture method based on aerial images for accurately estimating nest abundance of a colony-breeding bird species.

Our case study was the Malagasy pond heron (*Ardeola idae*), which was surveyed on the island of Mayotte. This Ardeidae species breeds in colonies on four islands in the Indian Ocean: Madagascar, Aldabra (Seychelles), Europa and Mayotte (France)^41,42^. Listed as an endangered species^43^, the Malagasy pond heron is threatened by habitat destruction and degradation (especially by the conversion of wetlands into agricultural areas), egg poaching, and rat predation on eggs and chicks^41,42^. Several populations, including on Mayotte, breed in mangroves^42^, an ecosystem particularly subject to anthropogenic pressure^44^. The world population of the species has recently been estimated at around 1100 breeding individuals^42^. Sharp population declines were reported in Madagascar between 1993 and 2016^42^, and on Aldabra between 1971 and 2001^45^. The vulnerability of this species makes it crucial to regularly reassess its conservation status by estimating its long-term population trends in order to recommend conservation measures^41,42^.

However, monitoring Malagasy pond heron populations is challenging, as many colonies are difficult to reach by foot and are located in high-density vegetation habitats such as mangroves, impeding good sightings of the nests and individuals from the ground^45^. Moreover, many are multispecies colonies, hosting other Ardeidae species such as the cattle egret *Bubulcus ibis*^41,46^, a similar-sized and mostly white species, which may lead to misidentification risks. Like many neotropical birds, the species has an extended breeding period^41^, and there may be asynchrony between breeding individuals, notably between colonies. While all these factors may lead to variation in detection of individuals and nests, they have not been considered in previous studies.

Images captured during aerial surveys by drones offer a potential way to address detectability issues when estimating breeding population size and trends of colonial bird species. They allow regular and detailed monitoring of colonies while minimizing disturbance. To assess the relevance of this method and the potential factors impacting detectability, we conducted repeated drone flights on Malagasy pond heron breeding colonies during the reproduction period. The study aims were to (i) identify daily and seasonal fluctuations of the abundance of adults and immature birds; (ii) estimate nest detection probability and abundance using capture-recapture methods; (iii) perform simulations to optimize the sampling design for detecting a decline in abundance.

## Methods

### Study area and species

**Figure 1:**
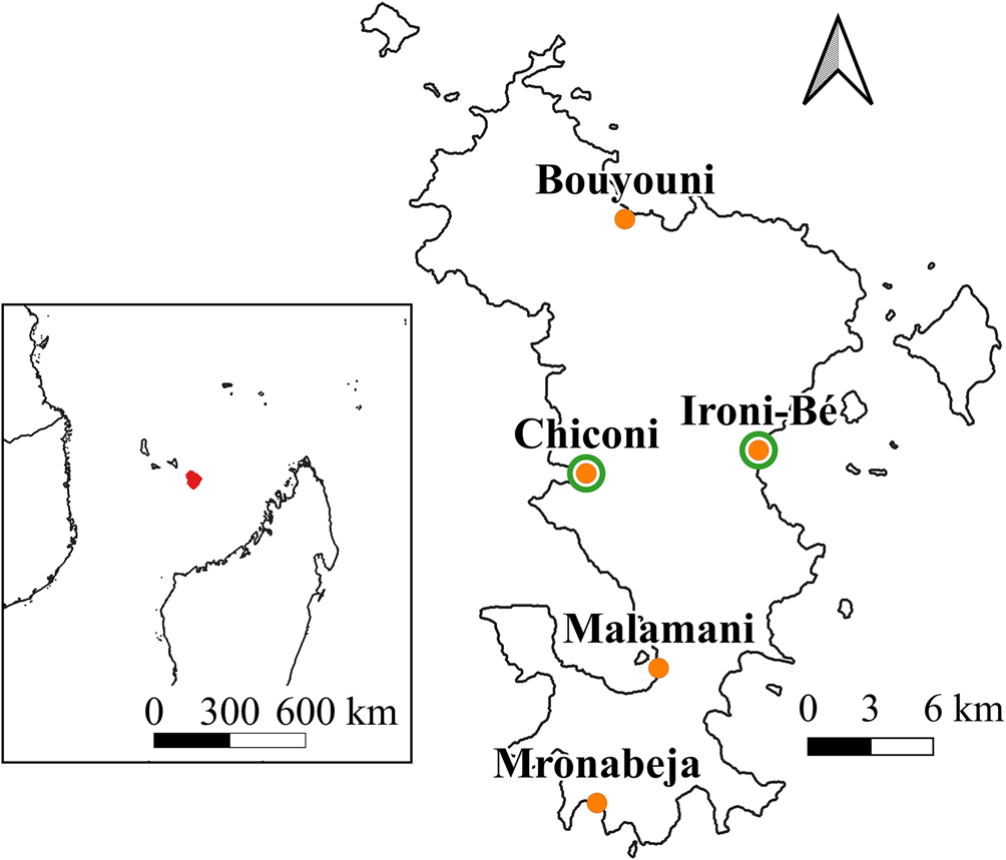
Location of the island of Mayotte (indicated in red in the inset panel) and of the breeding colonies of Malagasy pond herons sampled from October 2020 to January 2021 (green circles) and from October 2021 to November 2021 (orange dots) on Mayotte.

The study was conducted on Mayotte, a French island located in the Mozambique Channel (Fig. 1). Its land surface area is 374 km^2^, including 16 km^2^ of wetlands, of which mangroves have the highest avian richness^46^. These habitats are in decline due to an increase of agricultural land and urbanized areas on the coast^47^. Mangroves are also subject to other anthropogenic pressures such as pollution caused by agriculture and wastewater treatment issues^44^.

The Malagasy pond heron is 45–48 cm in length^48^. During the breeding period, adults are distinguished from other heron species by their snow-white plumage and azure-blue beak. In the inter-nuptial period, individuals are streaked with brown, beige and black, with white wings and a grey-green beak. The plumage of immature birds is similar to inter-nuptial birds, but with brown wingtips^41^. The breeding season of this southern hemisphere species starts in October and ends in March; during the breeding season colonies form in Madagascar and the nearby islands of Aldabra (Seychelles), Mayotte and Europa (France)^42,45,48^. These are generally mixed-species colonies, including the squacco heron *Ardeola ralloides* on Madagascar and the cattle egret on Mayotte^41,46^. Misidentification between Malagasy pond herons and cattle egrets may occur when individuals are only partially visible or images are blurred. Knowledge about Malagasy pond heron biology and ecology remains scarce^41,48^.

### Sampling design and data collection

The study surveyed the five known Malagasy pond heron breeding colonies on Mayotte (Fig. 1). During *year1*, we considered most of the breeding season period, with surveys occurring from October 2020 to January 2021. During *year2*, we focused on the beginning of the breeding season period, with surveys occurring from October to November 2021.

The first step was a flight by an ultralight aircraft over all the island’s mangroves on 11 September 2020 (*year1*) and 23 September 2021 (*year2*) to identify the establishment of colonies at the known locations (Fig. 1) and to detect potentially unknown colonies. A second ultralight aircraft flight was carried out on 3 December 2020 (*year1*) and 18 November 2021 (*year2*) to identify potential late colony settlements.

In the next step, a single drone operator conducted drone flyovers over the identified breeding colonies, using a DJI Mavic 2 Pro drone model with a 20MP L1D-20c sensor camera. The drone took off at a distance of more than 100 m from the colonies to minimize disturbance^34^. In previous years, several empirical flight tests had been conducted to define the flight parameters allowing the best compromise between the image resolution required to distinguish individuals and the flight height to minimize bird disturbance. The latter was assessed by the number of individuals taking flight during the drone approach or with beaks turned towards the drone camera. Based on these preliminary tests, the drone flights were carried out approximately 10 m above the canopy (between 15 and 20 m above the ground, depending on tree height) at a speed of 3 to 4 m/s. The flights lasted between 15 and 20 min, and 10 to 100 images were obtained for each of the five sampled colonies, depending on their spatial extent.

During *year1*, we selected two breeding colonies (Ironi-Bé and Chiconi, Fig. 1) with a large number of breeders compared to other colonies and with marked differences in canopy structure. Flyovers were conducted over these colonies six times a day: every two hours between sunrise and sunset. However, due to logistical constraints, some flyovers were delayed by around 30 min (see Table S1 in Supplementary Materials). These daily flyovers were repeated over three sessions: 15 and 22 October (session 1); 27 November and 4 December (session 2); and 15 and 22 January (session 3).

Simulations from the results obtained during *year1* indicated that at least three flyovers were required for optimal counts, so this was applied in *year2* (see the ‘Simulation’ section), during which flyovers were conducted over all five colonies (Fig. 1) once a day over three successive days. This daily flyover was repeated over three sessions: from 10 to 13 October (session 1); from 31 October to 3 November (session 2); from 21 to 23 November (session 3) (see Table S1 in Supplementary Materials). Due to logistical constraints, only two flyovers (separated by 3 days) were conducted at the Bouyouni colony during session 2.

### Image processing and identification of individuals and nests

After collecting the aerial images from *year1* and *year2*, we removed duplicates (i.e. almost identical images). We then assembled the images collected during one flyover, stitching multiple photos into a single image to avoid counting the same individual or nest more than once. For colonies located in dense and homogeneous canopies (e.g. Ironi-Bé and Bouyouni, Fig. 1), we used Autopano Giga 4.4, free software that automates panoramic image assembly, reducing data processing time. However, for colonies in mangroves with significant tree height variation, the automatic assembly of images taken from different angles did not work well. In these situations, we manually assembled the images using the free software GIMP (version 2.10.30). We did not georeference the images because the drone GPS was not accurate enough and we could not use any landmarks from the ground due to the mangrove vegetation.

From the resulting single images, we manually identified all Malagasy pond heron individuals and nests with QGIS software (version 3.16). We distinguished individuals in two categories, adults and immature birds. Immature birds included both chicks and juveniles born during the breeding season and birds born during the previous breeding season, these age categories being indistinguishable by plumage colour in drone images. For the nests, we excluded those occupied by cattle egrets, defining a pond heron-occupied nest as a nest with at least one adult or immature pond heron detected on it. We distinguished ‘occupied nests’ from ‘empty nests’ (i.e. absence of a distinguishable pond heron). For each occupied nest, we then recorded the number of immature birds, the number of adults and the number of eggs visible in the nest.

**Figure 2:**
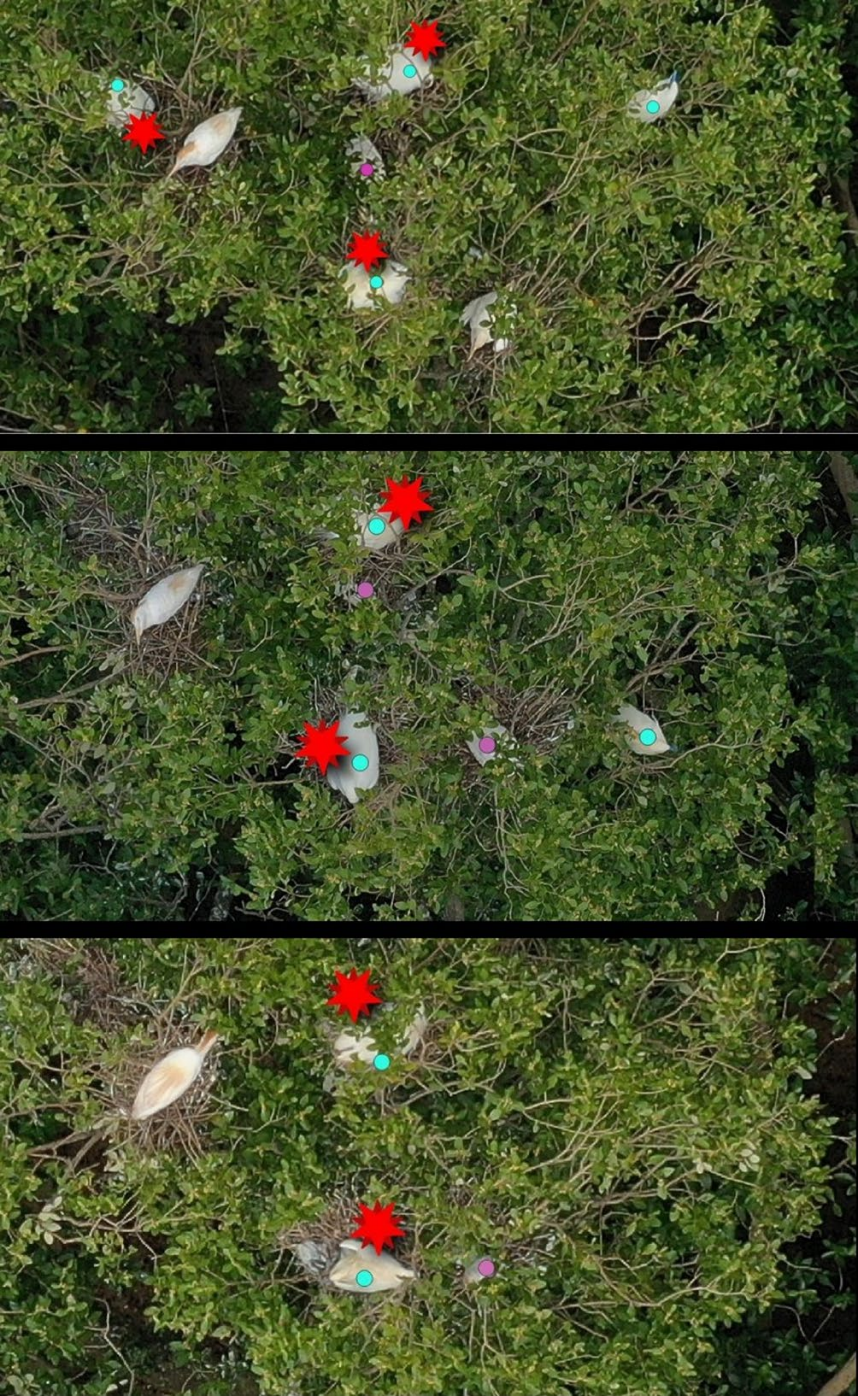
Zoom on the same area of aerial images obtained over three successive days (from top to bottom: 21–23 November 2021) by drone flyovers of a Malagasy pond heron colony. Blue dots are Malagasy pond herons and purple dots are unidentified individuals. Red stars are nests occupied by Malagasy pond herons detected by the image interpreter. Other visible herons are cattle egrets. During the first flyover, all the Malagasy pond heron nests were detected, whereas in the second and third flyovers, one nest was missed.

These image assembly and identification steps were conducted by different observers for each year (one in 2020–2021, another in 2021–2022). The identification step was performed colony after colony and then flyover after flyover to minimize the risks of memorizing the location of nests of the same colony from one image to another, which might violate the hypothesis of detection independence between the flyovers required in capture-recapture analysis^38^. For the capture-recapture data preparation (see ‘Data analysis’ section), we superimposed visually the images obtained over the successive times (*year1*) and days (*year2*) to match the same nests detected on the images from each session and identified the ones not detected on the other images from the session (Fig. 2). This was conducted only for the session (in each breeding season) with the maximum number of nests counted on an image from all the flyovers: we defined this session as the ‘reproduction peak session’. For *year1*, we attributed to each case of non-detection (n = 350) one of the following factors relating to detectability: (i) absence of individual on the active nest; (ii) active nest unavailable for detection due to image luminosity or the presence of tree branches; (iii) individual on the active nest not distinguishable from a cattle egret, and (iv) active nest occupied and visible, but missed by the observer. We also reported cases of active nests being cut during the image assembly, thus not sampled.

### Data analysis

#### Intra-daily and intra-seasonal variation in relative abundance (*year1*)

This analysis was based on individual counts and did not correct for imperfect detection. We analysed adult and immature counts in two breeding colonies (Ironi-Bé and Chiconi, Fig. 1) detected in images using generalized additive models (GAM) with Poisson distribution and a log link function. We included the session as a categorical fixed effect and the hour as a smooth term in interaction with the different sessions (categorical). We set the number of maximum smoothing parameters to k = 6, corresponding to the number of daily flyovers. The GAMs were fitted with the mgcv package^49^ in R^50^.

#### Detection probability *p* and abundance *N* estimates of active nests (*year1 and 2*)

We used closed capture models^31^ to estimate detection probability (*p*) and abundance (*N*) of active nests of Ironi-Bé and Chiconi breeding colonies (*year1*) and all the five breeding colonies (*year2*). For each year dataset, we gathered data from the different colonies and included the colonies as a group effect in the models to provide nest abundance estimates for each colony and test potential detection probability differences between them. We analysed data from the reproduction peak sessions (see ‘Results’) for *year1* and *year2* separately. One session data series corresponded to the six daily flyovers (*year1*), and the other to the one daily flyover on three successive days (*year2*). We hypothesized that the ‘nest population’ was closed (no appearance or disappearance of nests) within these closed sessions. We considered the active nests as those occupied by a pond heron during at least one flyover. The nest detection history for a flyover was 1 when an occupied nest was detected by the image interpreter and 0 when it was not detected. We fitted models with constant and time-dependent detection probability, and equal or different detection probability between the colonies additively. Of the four models tested, we selected the one with the lowest AIC score^51^. We performed these analyses with Mark (version 9.0) software, from R^50^ using the Rmark library^52^.

#### Capture–recapture data simulations to perform power tests

We used the mean detection probability of nests estimated by the constant model of capture–recapture for *year1* to perform power tests for detecting declines in abundance. We did not perform these power tests on increases in abundance as it is less of a priority for species in poor conservation status such as the Malagasy-pond heron. We simulated nest detection history with several variables for flyovers (ranging from 2 to 5) and colony size (50, 100, 150 and 200 nests). We ran 100 simulations for each of these combinations and calculated the mean coefficient variations (cv) of the nest abundance estimates. We also calculated the proportion of cases where the 95% confidence interval did not include a decrease in abundance of 1% to 20% and considered situations where this proportion exceeded 80% as situations with good power to detect decline.

## Results

### Intra-daily and intra-seasonal variation in relative abundance (year1)

**Figure 3:**
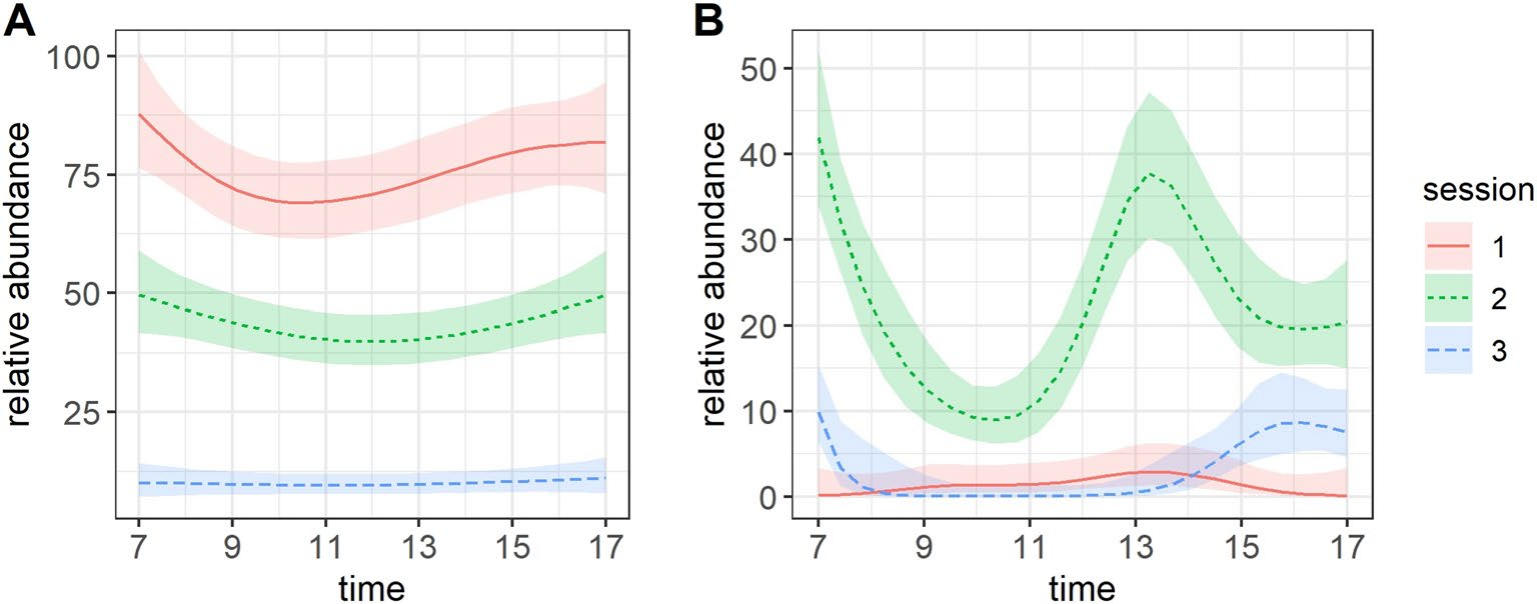
Relative abundance predictions (95% CI in shaded area) of adults (**A**) and immature birds (**B**) obtained from GAM models fitted to pond heron counts in two breeding colonies obtained from drone images collected during the 2020–2021 breeding season on Mayotte.

The GAM fitted on repeated counts revealed a high and significant decrease of Malagasy pond heron adult relative abundance throughout the sessions, whereas immature bird abundance was significantly higher during session 2 (end of November—beginning of December) than in sessions 1 (mid- to end-October) and 3 (mid- to end-January) (Fig. 3). The model predictions showed a slight decrease in adult abundance in late morning (session 1) and in the middle of the day (session 2). Immature bird abundance significantly dropped from 08:00 to 12:00 and after 14:00 during session 2.

### Intra-daily detection probability variation in active nests and causes of non-detection (year1)

**Figure 4:**
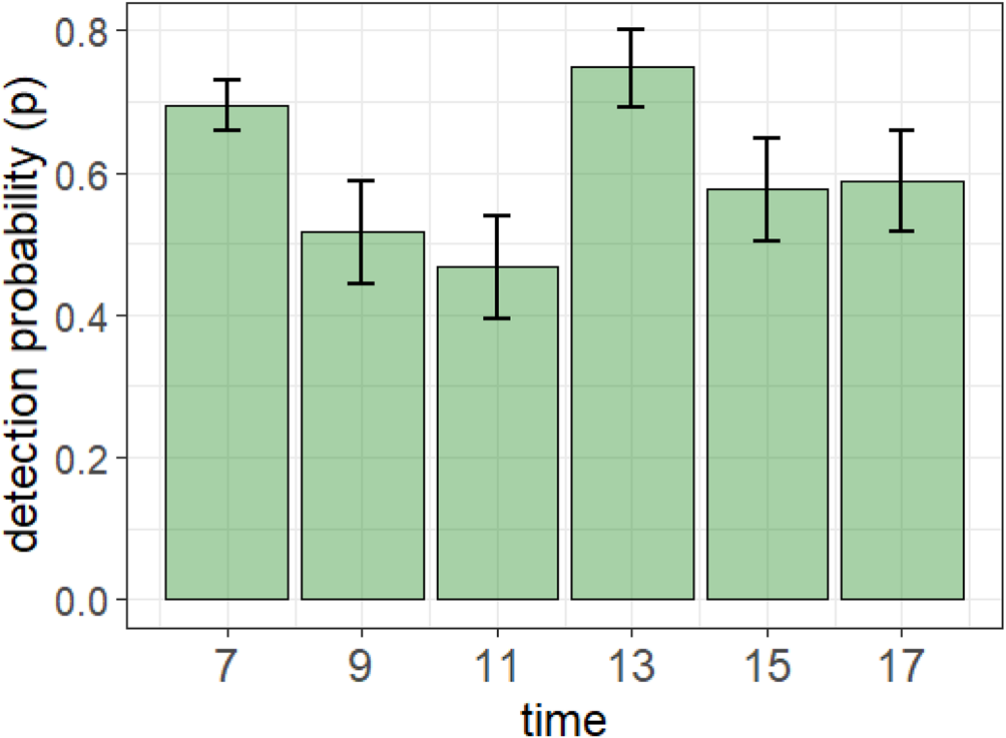
Detection probability *p* estimates (and their 95% CI) of active nests obtained from the capture-recapture time-dependent model fitted from data obtained from drone image analysis collected in two breeding colonies during the 2020–2021 breeding season on Mayotte*.*

The mean detection probability estimate obtained by the closed capture-recapture analysis was *p* = 0.60 [95% CI: 0.57–0.63] when considering the constant model. The detection probability did not differ between Ironi-Bé and Chiconi colonies. The best model was time-dependent. It estimated a *p* amplitude of 0.28, ranging from *p* = 0.47 [95% CI: 0.39–0.54] to *p* = 0.69 [95% CI: 0.62–0.76] depending on the time of the flyovers. The lowest *p* values were obtained for 9:00 and 11:00 (Fig. 4).

Regarding the causes of non-detection, 20% of undetected nests were due to the absence of a Malagasy pond heron on it, 33% were not detected due to image luminosity or the presence of tree branches, and 31% were due to individuals on the active nest not distinguished from cattle egrets. A further 11% of active nests occupied by a Malagasy pond heron were missed by the image interpreter. The remaining 5% of active nests were cut during image assembly.

### Abundance estimates of active nests on Mayotte colonies during the reproduction peak sessions (year2)

The reproduction peak sessions provided by the raw data differed between the colonies. For one colony, it occurred during session 1, for two colonies during session 2, and for another two colonies during session 3. We identified 403 different nests on all five colonies during the three flyovers of the reproduction peaks of each colony.

**Figure 5:**
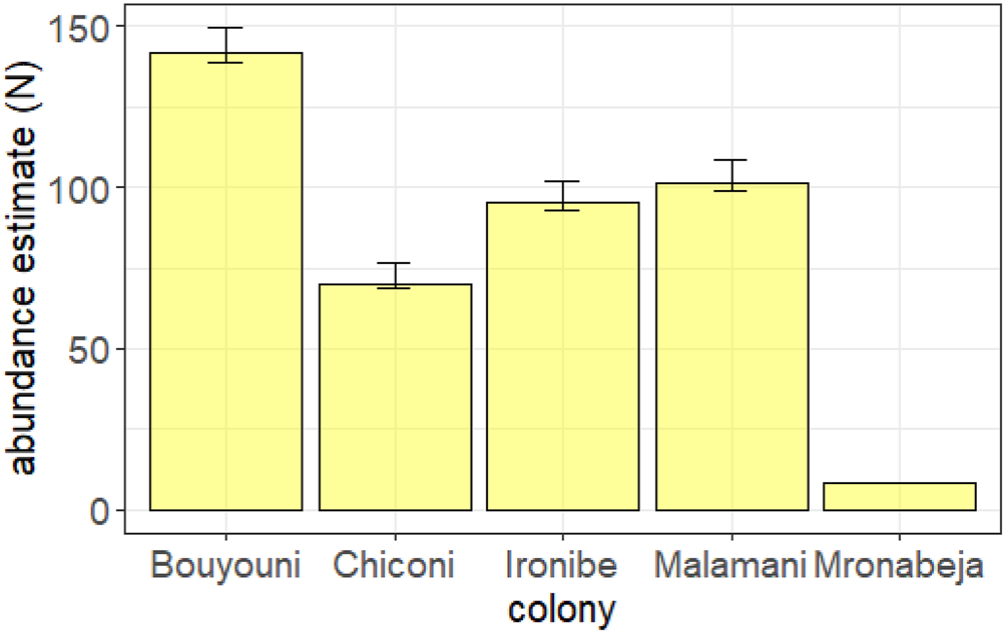
Abundance estimates (and 95% CI) of active nests obtained from the capture-recapture time-dependent model fitted from data of drone image analysis collected on five breeding colonies during the reproduction peak sessions of the 2021–2022 breeding season on Mayotte*.*

The closed capture-recapture analysis conducted on the data from those reproduction peak sessions revealed that the model with the lowest AIC score (ΔAIC = 5 with the subsequent model) was time-dependent, with no differences between colonies regarding ‘active nest’ detection probability *p*. This model estimated some detection probability variation between the flyovers: *p* = 0.66 [95% CI 0.61–0.70] for flyover 1; *p* = 0.74 [95% CI 0.69–0.78] for flyover 2; and *p* = 0.58 [95% CI 0.53–0.63] for flyover 3. The colony abundance estimates ranged from *N* = 8 [95% CI: 8–8] to *N* = 142 [95% CI: 139–150] active nests (Fig. 5). When considering the constant model, the mean detection probability estimate was *p* = 0.66 [95% CI: 0.63–0.69].

### Capture–recapture dataset simulations to perform power tests

**Figure 6:**
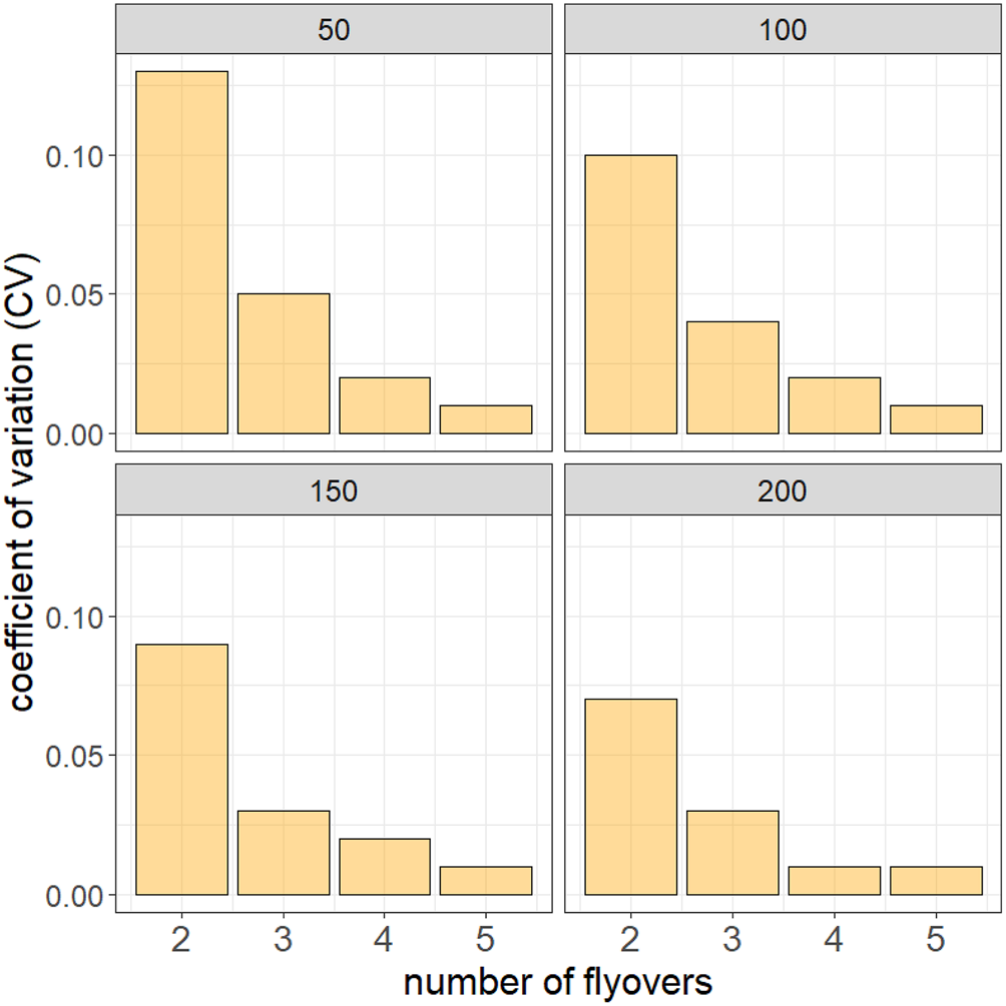
Mean coefficients of variation (cv) calculated from 100 simulations of nest capture histories with detection probability *p* = 0.60 for colony sizes ranging from 50 to 200 nests.

The mean coefficient of variation (cv) in abundance obtained from simulations was high for two flyovers, especially for small colonies (50 individuals) (cv = 0.13) (Fig. 6). Such a high cv did not allow the detection of a decline in abundance lower than 16% (see Table S2 in Supplementary Materials). This cv decreased sharply for three flyovers, with cv = 0.05 for colonies of less than 50 individuals and cv < 0.05 for colonies of more than 50 individuals. These lower cvs allow the detection of a decline in abundance as low as 5% to 6% depending on the colony size. When considering four flyovers, cv < 0.03 whatever the size of the colony, allowing the detection of a decline in abundance of 2% to 3%.

## Discussion

This study took an original approach to investigate daily and seasonal variations in abundance and nest detectability of a colonial bird species based on aerial images obtained from drone flights. The approach allowed us to obtain unbiased estimates of nest abundance using closed capture-recapture models. The estimates showed mean detection probabilities of active nests of 0.60 and 0.66 depending on years; this means that the aerial census was far from exhaustive, as 34% to 40% of the nests were not detected during just one flyover. The findings also show that nest detection probability varied temporally as a consequence of several detectability factors^16,17^. Raw counts of nest abundance based on aerial images alone thus underestimate nest abundance and cannot be compared between sites or over time due to variation in detection probability.

The repeated flyovers showed high seasonal and intra-daily variation in relative abundance of Malagasy pond heron individuals detected at the colonies. These variations may be related to the first component of the detection process, i.e. the probability that individuals are present in the colony during the drone flyover. The peak of abundance of adults recorded between mid- and end-October during the 2020–2021 breeding season corresponds to the settlement of pairs in a colony, with the construction of nests and the first laying and brooding^41^. During this period, we recorded only a few immature birds, probably corresponding to the very first young to hatch whose size was large enough to be detectable from aerial images. We also detected a slight decrease in adult abundance in the colonies in late morning, probably due to foraging activity.

Between late November and early December, the number of adults simultaneously present in the colony decreased and stayed fairly constant during the day, likely due to time spent by one of the individuals in a pair in foraging activity out of the colony. During the same period, the number of immature birds detected in the images increased as the young developed. However, their relative abundance varied during the day, decreasing sharply during mid-morning and the end of the afternoon. For immature birds that have not yet begun to fly (i.e. chicks), the decrease in abundance in this age class could be due to behavioural changes during the breeding season. For instance, as they become more mobile, they could leave the nest and hide under the canopy to avoid heat, direct sunlight or predators, making them unavailable to detection in aerial images. In contrast, birds over one year old that have been flying for some time could leave the colony to reach other resting or feeding sites during the day. Between mid to late January, adult and immature bird abundance decreased due to the higher autonomy of chicks requiring less adult attending, as well as to juvenile dispersal out of the nests, or even out of the colonies. In our study, this period, which corresponded to the end of reproduction season, was two months earlier than the one reported in another study^41^.

As daily and seasonal variation in the relative abundance of Malagasy pond herons in the colonies affect the first component of the detection process, this in turn strongly impacts the availability of active nests for detection (the second component of the detection process) since we used individuals to detect nests. We estimated that nests unoccupied by individuals were the cause of up to 20% of non-detection of the active nests detected at least once by the image interpreter. This was confirmed by our capture-recapture study in *year1*, which revealed that nest detection probability decreased about 25% from 7:00 to 11:00, a similar pattern as recorded for adult relative abundance variation during the first aerial session and for immature bird abundance variation during the second aerial session. In addition to the absence of individuals on active nests, the second component of the detection process was affected by other phenomena. One third of non-detection of nests was attributable to luminosity or vegetation such as tree branches masking the nests, as observed in another study^15^.

Regarding the third component of the detection process, we estimated that 11% of active nests occupied by individuals were missed by the image interpreter. Additionally, one-third of non-detection of nests was due to individuals not being distinguished from cattle egrets. Misidentification issues from aerial images have also been reported for other waterbird species^23,26^. These observer bias could be investigated through multiple observers experiments on single images to test for potential differences between observers. However, such an approach is not sufficient to estimate the overall detection process as only the nests available to detection (and with an individual identifiable on it) would be considered into the analysis. Instead, the capture-recapture approach we used integrates the three components of the detection process. The detection probabilities we estimated with this approach were quite similar between years, suggesting that the change of observer did not affect this parameter.

These results show that correcting for detection issues is key in order to obtain unbiased estimates of nest abundance. In theory, this would involve conducting repeated censuses over a short time interval to use a closed capture-recapture approach. Yet multiple flyovers might increase the risks of disturbance of individuals in colonial bird species. It is thus crucial to find the right balance between estimate accuracy and frequency of drone flights in accordance with the study goal. To estimate abundance, double-count methods^15,24,37^ may be suitable for dealing with imperfect detection of objects, like the active nests considered here. However, the power tests based on the mean nest detection probability we estimated showed that two counts provide fairly unprecise abundance estimates. Three counts (in our case, flyovers) provided more accurate estimations of active nest abundance for Malagasy pond heron colonies of 50 to 200 pairs, allowing the detection of a decline in abundance as small as 5–6%. These three flyovers should be spaced at most a few days apart to fulfil the closure assumption of closed capture–recapture models.

The aerial surveys should preferentially be conducted during early morning and early afternoon, as nest detection probability was higher at these times. As the peak period of abundance differed between colonies during the second year, and may also vary between years, we recommend conducting three sessions (of three flyovers each) spread over the entire breeding season and retaining the maximum abundance value to estimate population abundance trends. Finally, as many previous studies have advocated in assessing perturbation risks when using drones^34,35^, we recommend running pilot studies to get a rough estimate of detection probability of the target species/colony on which to build power tests to develop optimized sampling designs.

Even in the context of rapid advances in technology and image processing, detectability will remain an issue in population monitoring. It is true that digital cameras and image analysis are rapidly evolving^3,4,8,10,53^, and higher image resolution should increase detectability of active nests by increasing the visibility of individuals^6^ and reducing misidentification errors with other species. There have been advances in automated counting and computer vision/machine learning approaches for monitoring target species from aerial images in recent years^2,4,7,54^, which have been tested successfully on large bird colonies aggregating thousands of individuals^3,8,53^. Nonetheless, while these techniques can reduce image analysis time^4^, they do not necessarily increase detection probability compared to manual counts^7^. Drones could also be equipped with infrared cameras or combine dual visible-thermal imagining cameras^54,55^ to increase the visibility of active nests under the canopy that are difficult to detect by image interpreters. This approach has been proven to increase the detection of individuals such as mammals^56^ or nests of cryptic waterbirds in complex vegetation structures^57^. Yet even if all these technical improvements increase the detectability of individuals and nests, they will not take into account the probability that an individual bird is present in the colony when the flyover is performed. An increase in detection probability over the years will additionally introduce a new source of variation that may lead to biased estimates of population trends if not considered. We thus strongly advocate for dealing with detectability issues in aerial counts by explicitly modelling detection probability using methods such as capture-recapture, especially for monitoring population trends of colonial birds.

### Supplementary Information

Supplementary Table S1.

Supplementary Table S2.

## Data Availability

Data and analysis scripts used in this research are available on Zenodo at https://zenodo.org/deposit/8403140 (doi: 10.5281/zenodo.8403140).
