# Supplementary material for: Airborne imagery does not preclude detectability issues in estimating bird colony size
Source: Sci Rep. 2024 Feb 14;14:3673. doi: 10.1038/s41598-024-53961-w (PMC10864377; doi:10.1038/s41598-024-53961-w)
Supplement: Supplementary file 1 — Supplementary Table S1. [file 41598_2024_53961_MOESM1_ESM.pdf]

Table S1: Dates and times of drone flights conducted during year 1 and 2 to obtain the data used for the analysis

colony	sampling year	date	flight number	time
Ironi-Bé	1	15/10/2020	1	07:30
Ironi-Bé	1	15/10/2020	2	09:30
Ironi-Bé	1	15/10/2020	3	11:30
Ironi-Bé	1	15/10/2020	4	13:30
Ironi-Bé	1	15/10/2020	5	15:30
Ironi-Bé	1	15/10/2020	6	17:30
Chiconi	1	22/10/2020	1	07:30
Chiconi	1	22/10/2020	2	09:30
Chiconi	1	22/10/2020	3	11:30
Chiconi	1	22/10/2020	4	13:30
Chiconi	1	22/10/2020	5	15:30
Chiconi	1	22/10/2020	6	17:30
Ironi-Bé	1	27/11/2020	1	07:30
Ironi-Bé	1	27/11/2020	2	09:30
Ironi-Bé	1	27/11/2020	3	11:30
Ironi-Bé	1	27/11/2020	4	13:30
Ironi-Bé	1	27/11/2020	5	15:17
Ironi-Bé	1	27/11/2020	6	17:00
Chiconi	1	04/12/2020	1	07:00
Chiconi	1	04/12/2020	2	09:00
Chiconi	1	04/12/2020	3	11:00
Chiconi	1	04/12/2020	4	13:00
Chiconi	1	04/12/2020	5	15:00
Chiconi	1	04/12/2020	6	17:00
Ironi-Bé	1	15/01/2021	1	07:30
Ironi-Bé	1	15/01/2021	2	09:30

Ironi-Bé	1	15/01/2021	3	11:30
Ironi-Bé	1	15/01/2021	4	13:30
Ironi-Bé	1	15/01/2021	5	15:30
Ironi-Bé	1	15/01/2021	6	17:30
Chiconi	1	22/01/2021	1	07:30
Chiconi	1	22/01/2021	2	09:30
Chiconi	1	22/01/2021	3	11:30
Chiconi	1	22/01/2021	4	13:30
Chiconi	1	22/01/2021	5	15:30
Chiconi	1	22/01/2021	6	17:30
Bouyouni	2	11/10/2021	1	06:32
Bouyouni	2	12/10/2021	2	08:48
Bouyouni	2	13/10/2021	3	14:43
Chiconi	2	21/11/2021	1	08:10
Chiconi	2	22/11/2021	2	11:24
Chiconi	2	23/11/2021	3	12:24
Ironi-Bé	2	31/10/2021	1	08:51
Ironi-Bé	2	01/11/2021	2	09:58
Ironi-Bé	2	02/11/2021	3	14:20
Malamani	2	31/10/2021	1	10:25
Malamani	2	01/11/2021	2	08:20
Malamani	2	02/11/2021	3	16:25
Mronabeja	2	21/11/2021	1	16:04
Mronabeja	2	22/11/2021	2	09:21
Mronabeja	2	24/11/2021	3	15:58
