# Supplementary material for: Airborne imagery does not preclude detectability issues in estimating bird colony size
Source: Sci Rep. 2024 Feb 14;14:3673. doi: 10.1038/s41598-024-53961-w (PMC10864377; doi:10.1038/s41598-024-53961-w)
Supplement: Supplementary file 2 — Supplementary Table S2. [file 41598_2024_53961_MOESM2_ESM.pdf]

Table S2: Results of simulations showing the mean coefficient of variation (cv) and the percentage of cases in which abundance was under the 95% CI interval limit for several rates of decline ranging from 1% to 20%, colony sizes in number of nests (N) ranging from N=50 to N=200, and number of drone flights (k) ranging from k=2 to k=5. Red: the exceeding threshold of 80% considered as a good power to detect decline.

N	k	mean cv	-1%	-2%	-3%	-4%	-5%	-6%	-7%	-8%	-9%	-10%	-11%	-12%	-13%	-14%	-15%	-16%	-17%	-18%	-19%	-20%
50	2	0.13	0.05	0.07	0.10	0.15	0.24	0.31	0.38	0.45	0.52	0.58	0.60	0.63	0.71	0.77	0.79	0.86	0.89	0.92	0.92	0.93
100	2	0.10	0.01	0.01	0.02	0.07	0.14	0.23	0.27	0.42	0.53	0.59	0.64	0.70	0.74	0.81	0.86	0.89	0.90	0.91	0.91	0.93
150	2	0.09	0.00	0.00	0.00	0.03	0.10	0.18	0.25	0.32	0.43	0.53	0.65	0.71	0.79	0.84	0.87	0.90	0.94	0.95	0.97	0.98
200	2	0.07	0.00	0.00	0.00	0.01	0.07	0.17	0.35	0.50	0.60	0.68	0.83	0.89	0.92	0.95	0.97	0.97	0.98	0.98	0.98	0.99
50	3	0.05	0.09	0.26	0.50	0.60	0.73	0.85	0.87	0.93	0.95	0.96	0.98	0.98	0.98	0.98	0.98	0.99	0.99	0.99	0.99	1.00
100	3	0.04	0.00	0.09	0.37	0.54	0.74	0.85	0.96	0.98	0.99	1.00	1.00	1.00	1.00	1.00	1.00	1.00	1.00	1.00	1.00	1.00
150	3	0.03	0.00	0.09	0.37	0.68	0.89	0.98	1.00	1.00	1.00	1.00	1.00	1.00	1.00	1.00	1.00	1.00	1.00	1.00	1.00	1.00
200	3	0.03	0.00	0.05	0.35	0.68	0.89	0.97	1.00	1.00	1.00	1.00	1.00	1.00	1.00	1.00	1.00	1.00	1.00	1.00	1.00	1.00
50	4	0.02	0.50	0.77	0.85	0.93	0.98	0.99	1.00	1.00	1.00	1.00	1.00	1.00	1.00	1.00	1.00	1.00	1.00	1.00	1.00	1.00
100	4	0.02	0.29	0.76	0.92	0.98	1.00	1.00	1.00	1.00	1.00	1.00	1.00	1.00	1.00	1.00	1.00	1.00	1.00	1.00	1.00	1.00
150	4	0.02	0.11	0.82	0.99	1.00	1.00	1.00	1.00	1.00	1.00	1.00	1.00	1.00	1.00	1.00	1.00	1.00	1.00	1.00	1.00	1.00
200	4	0.01	0.07	0.84	1.00	1.00	1.00	1.00	1.00	1.00	1.00	1.00	1.00	1.00	1.00	1.00	1.00	1.00	1.00	1.00	1.00	1.00
50	5	0.01	0.92	0.97	1.00	1.00	1.00	1.00	1.00	1.00	1.00	1.00	1.00	1.00	1.00	1.00	1.00	1.00	1.00	1.00	1.00	1.00
100	5	0.01	0.90	1.00	1.00	1.00	1.00	1.00	1.00	1.00	1.00	1.00	1.00	1.00	1.00	1.00	1.00	1.00	1.00	1.00	1.00	1.00
150	5	0.01	0.89	1.00	1.00	1.00	1.00	1.00	1.00	1.00	1.00	1.00	1.00	1.00	1.00	1.00	1.00	1.00	1.00	1.00	1.00	1.00
200	5	0.01	0.94	1.00	1.00	1.00	1.00	1.00	1.00	1.00	1.00	1.00	1.00	1.00	1.00	1.00	1.00	1.00	1.00	1.00	1.00	1.00
